# Supplementary material for: Detection of Oxazolidinone Resistance Genes and Characterization of Genetic Environments in Enterococci of Swine Origin, Italy
Source: Microorganisms. 2020 Dec 17;8(12):2021. doi: 10.3390/microorganisms8122021 (PMC7766396; doi:10.3390/microorganisms8122021)
Supplement: Supplementary file 1 [file microorganisms-08-02021-s001.zip › Figure S2.pptx]

## Slide 1
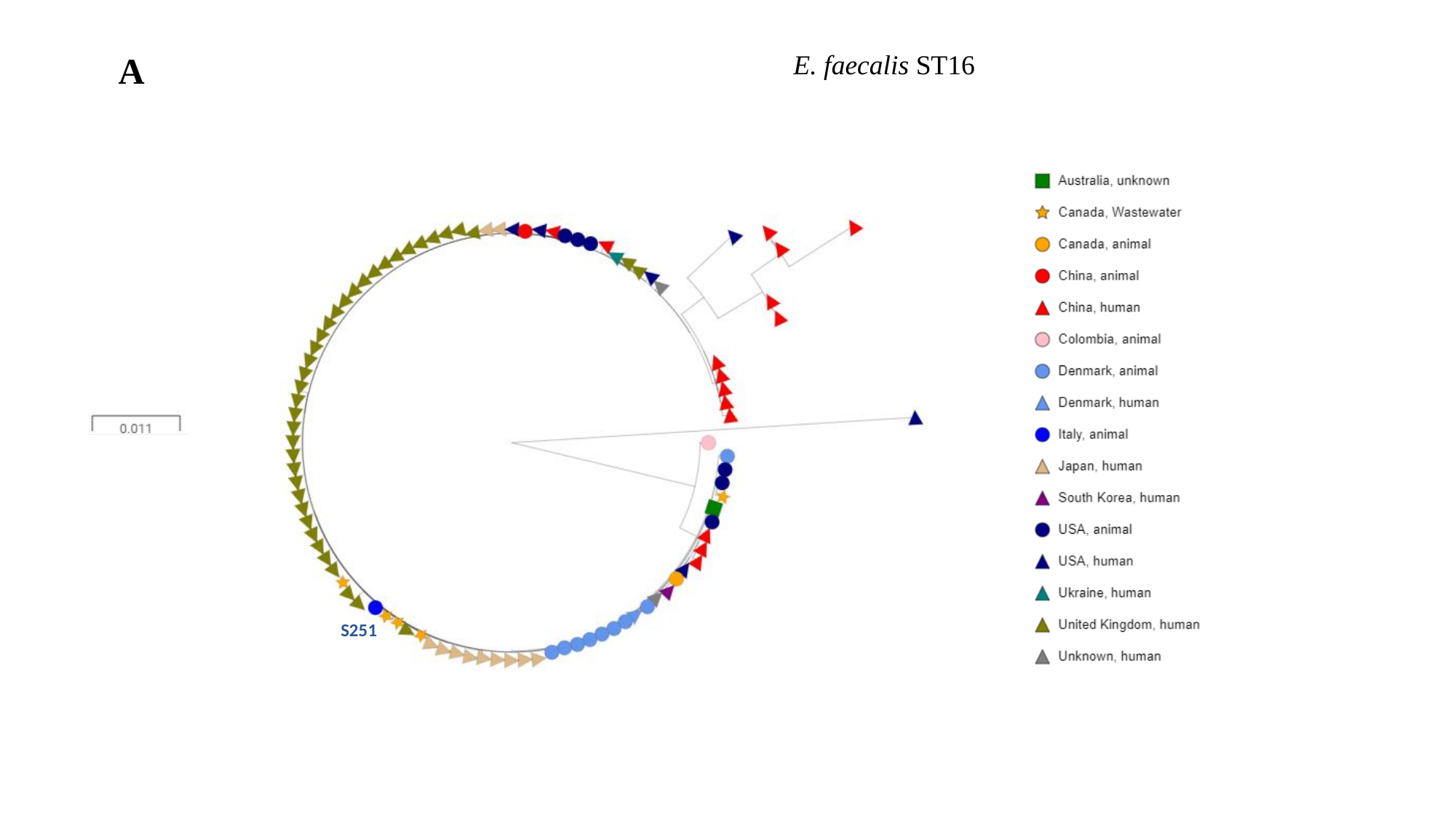

E. faecalis ST16
S251
A

## Slide 2
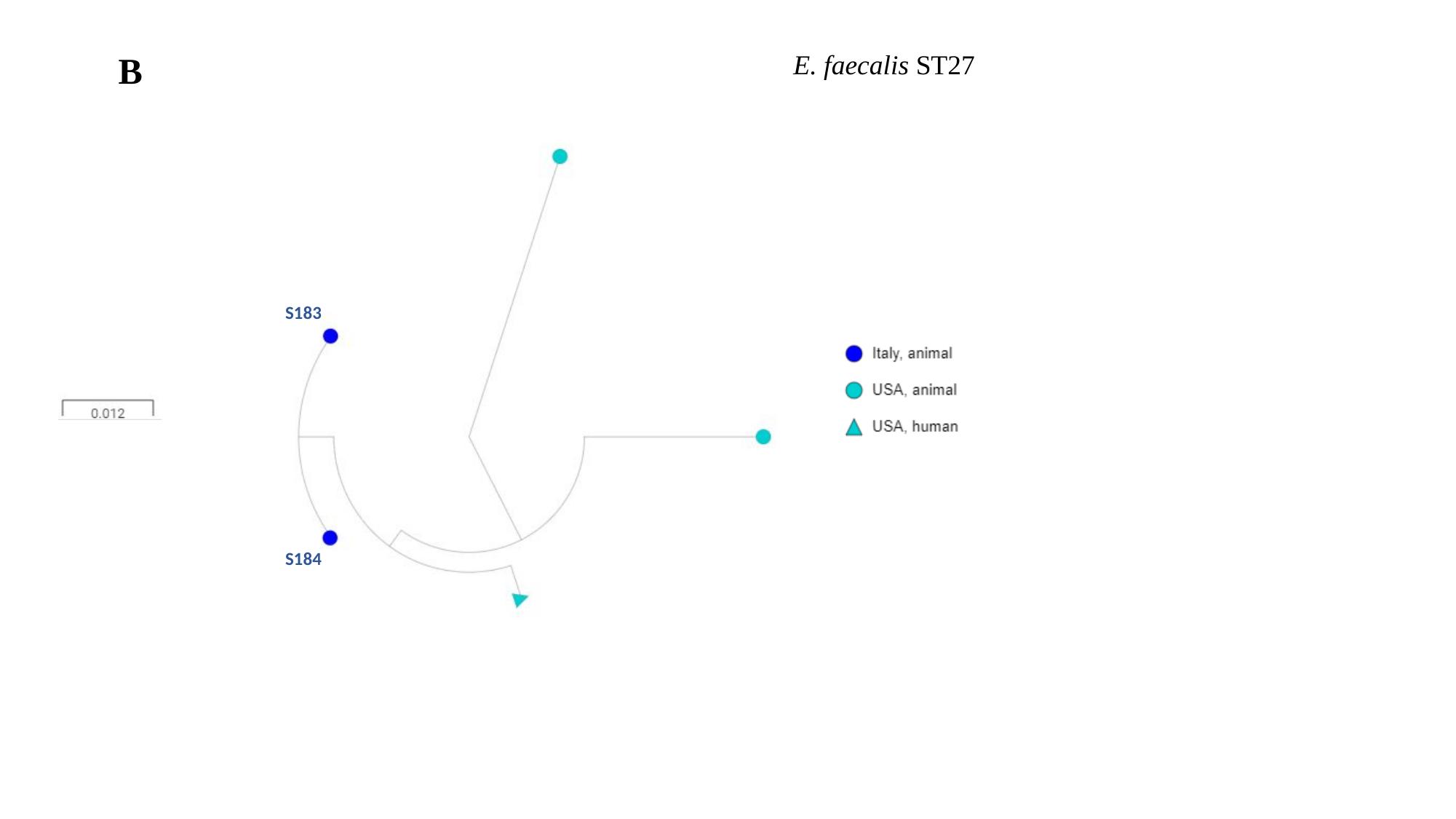

E. faecalis ST27
B
S183
S184

## Slide 3
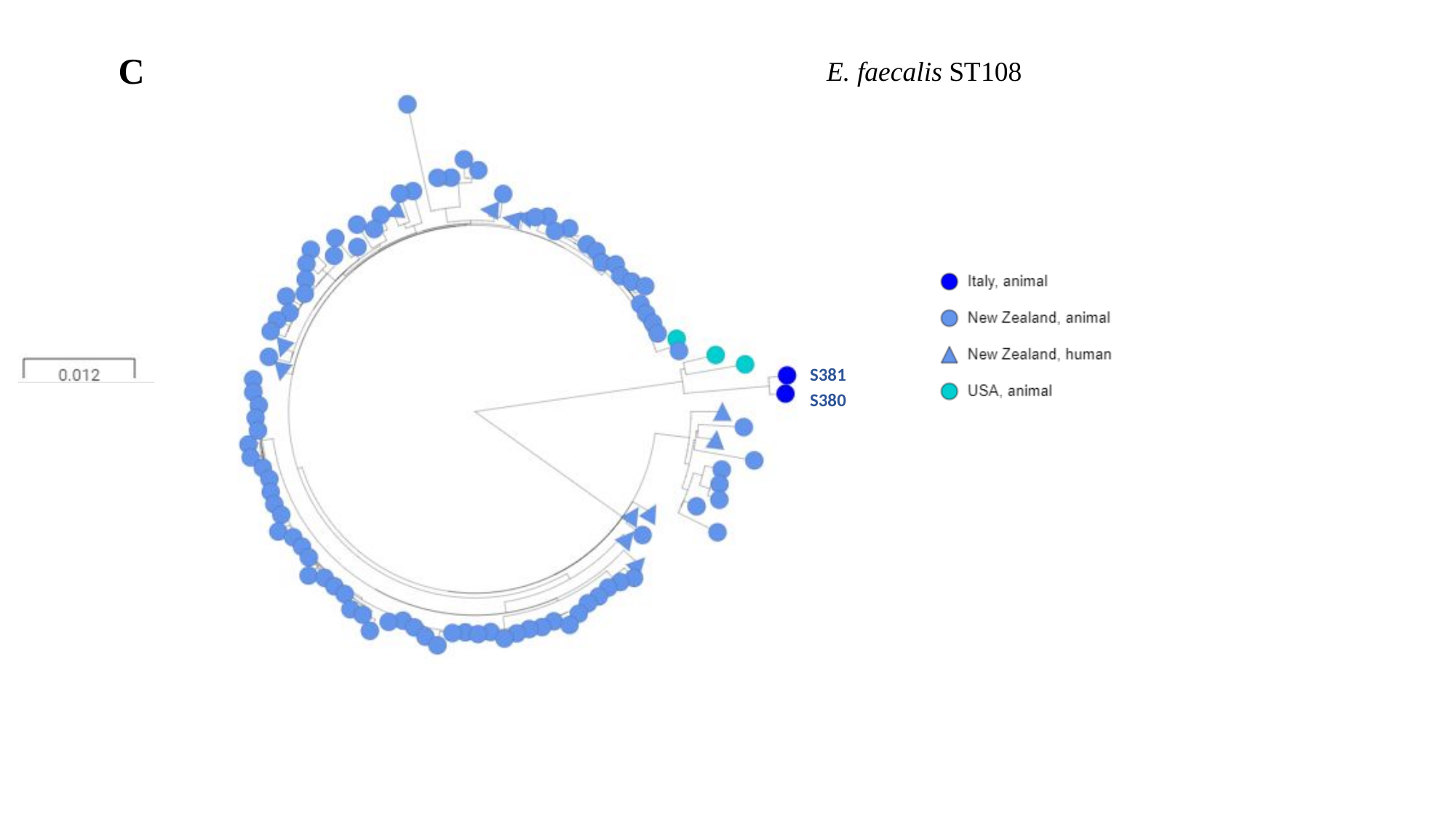

C
E. faecalis ST108
S381
S380

## Slide 4
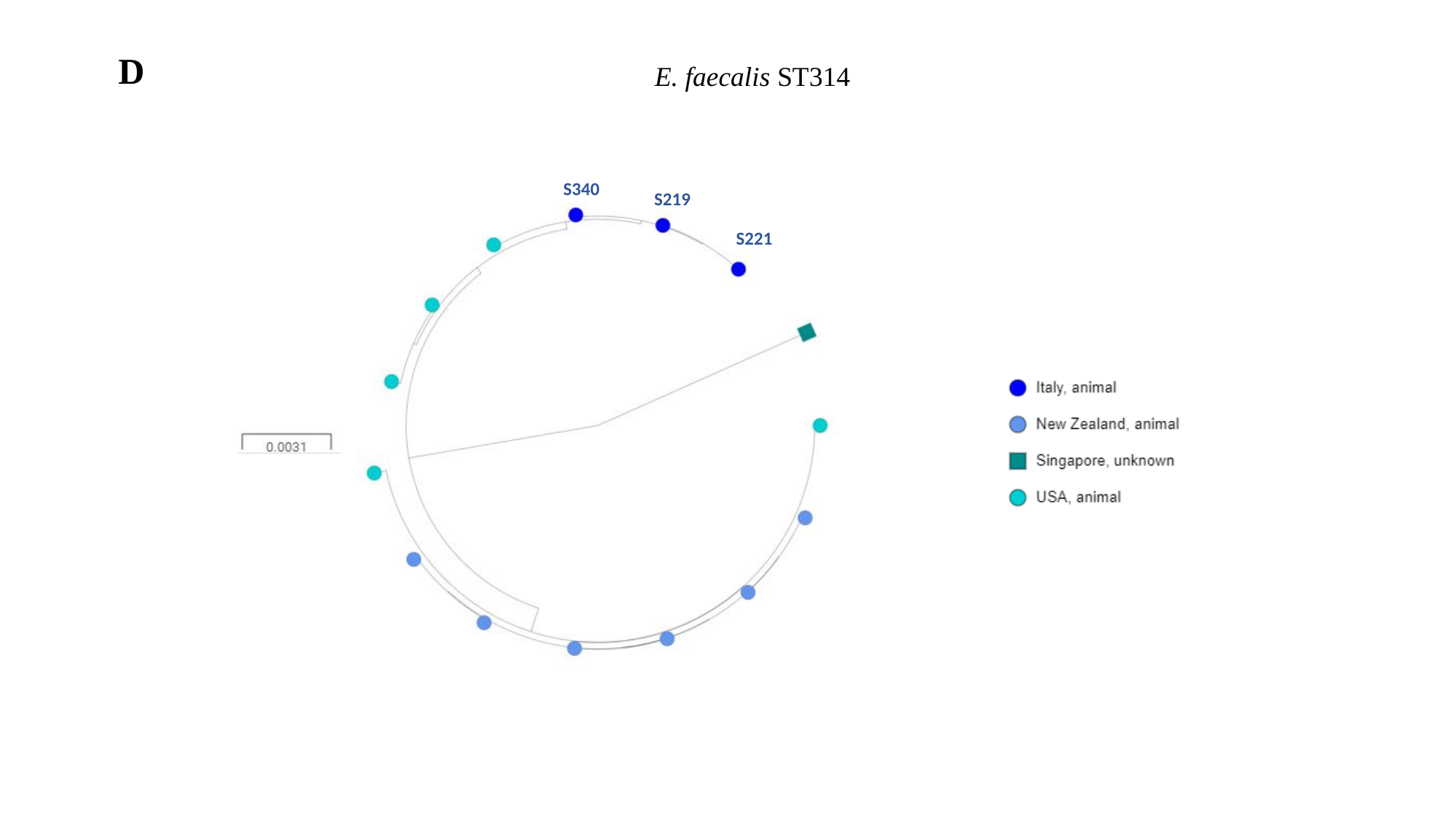

D
E. faecalis ST314
S340
S219
S221

## Slide 5
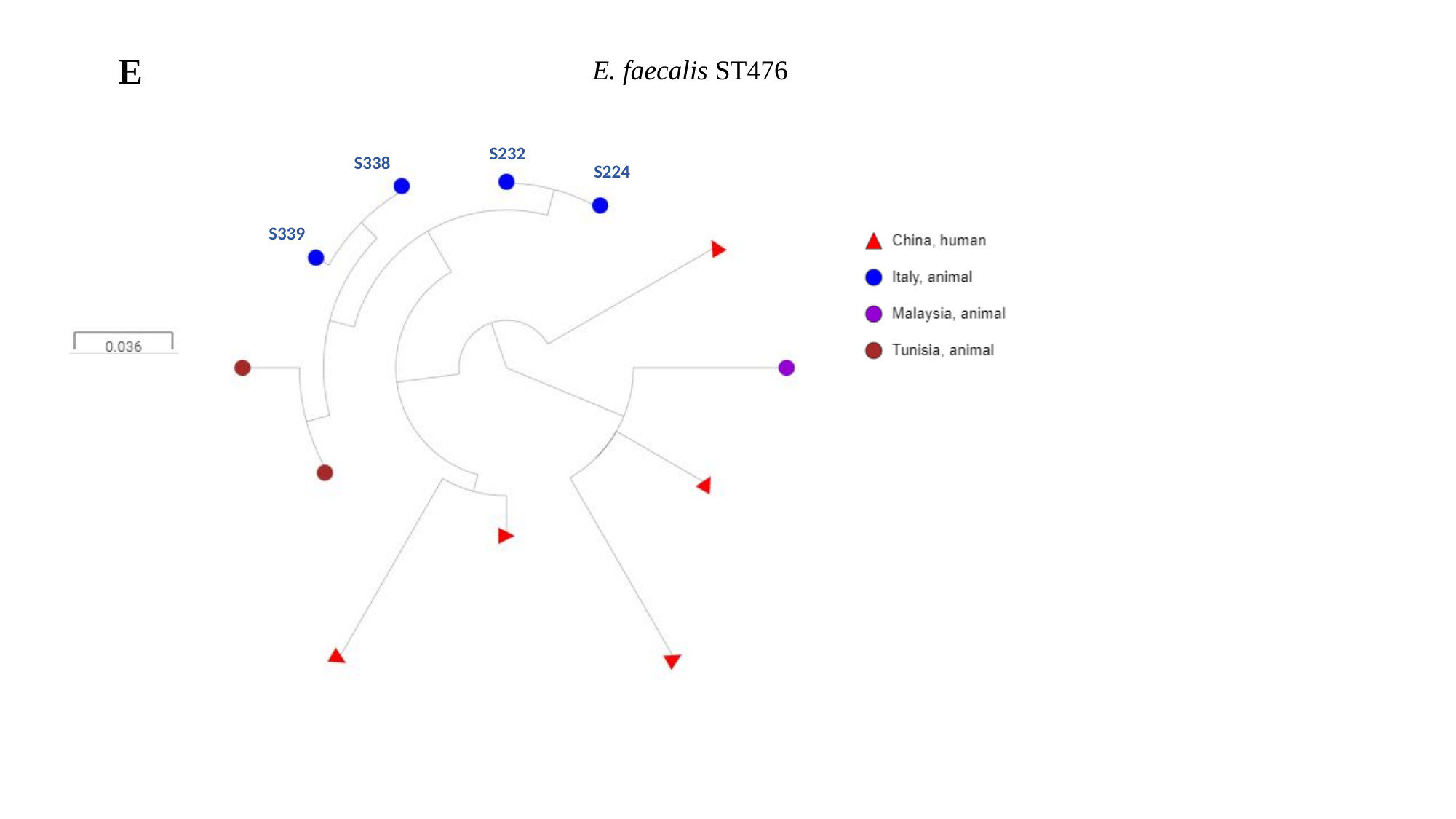

E
E. faecalis ST476
S232
S338
S224
S339

## Slide 6
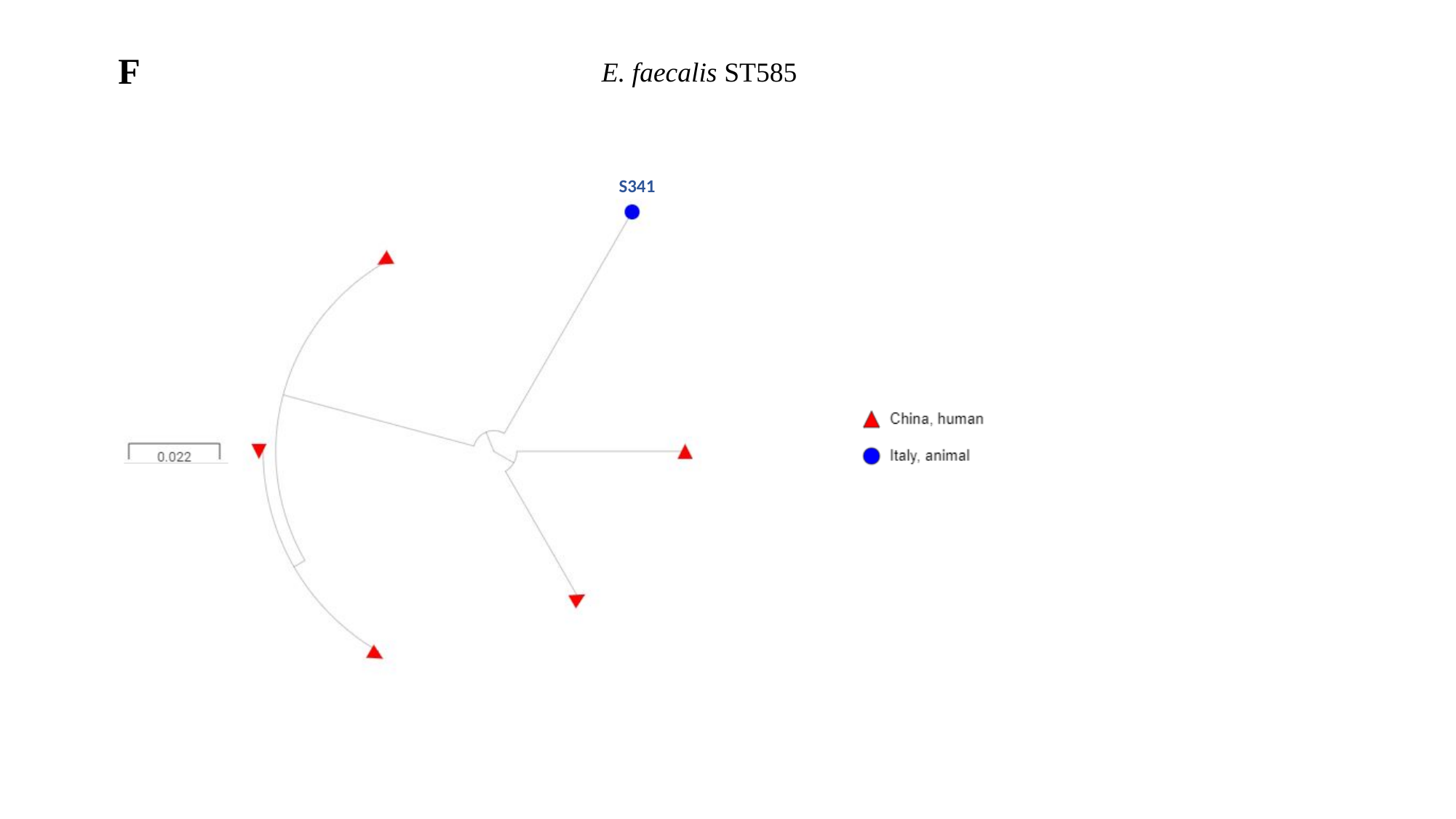

F
E. faecalis ST585
S341

## Slide 7
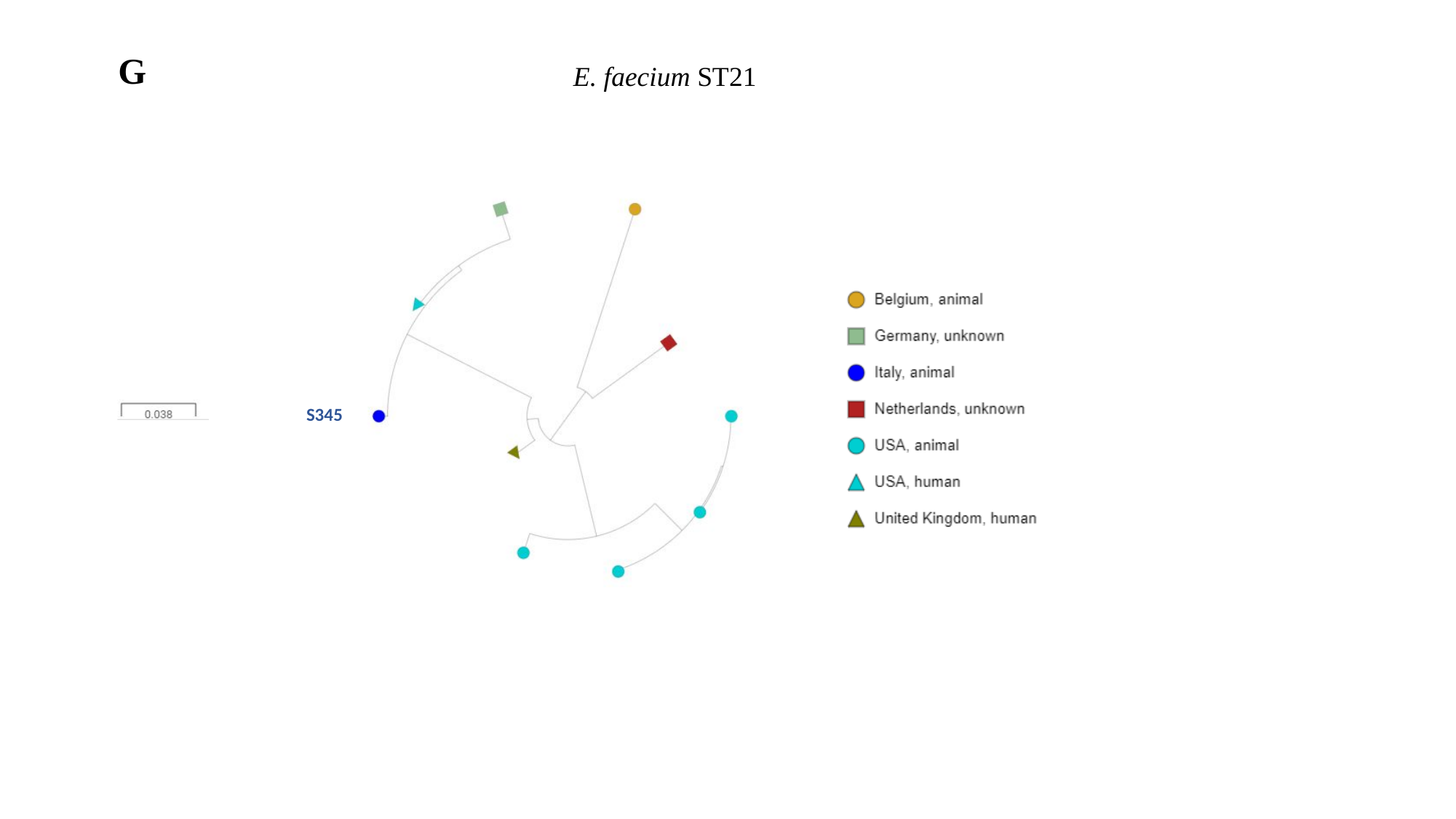

G
E. faecium ST21
S345

## Slide 8
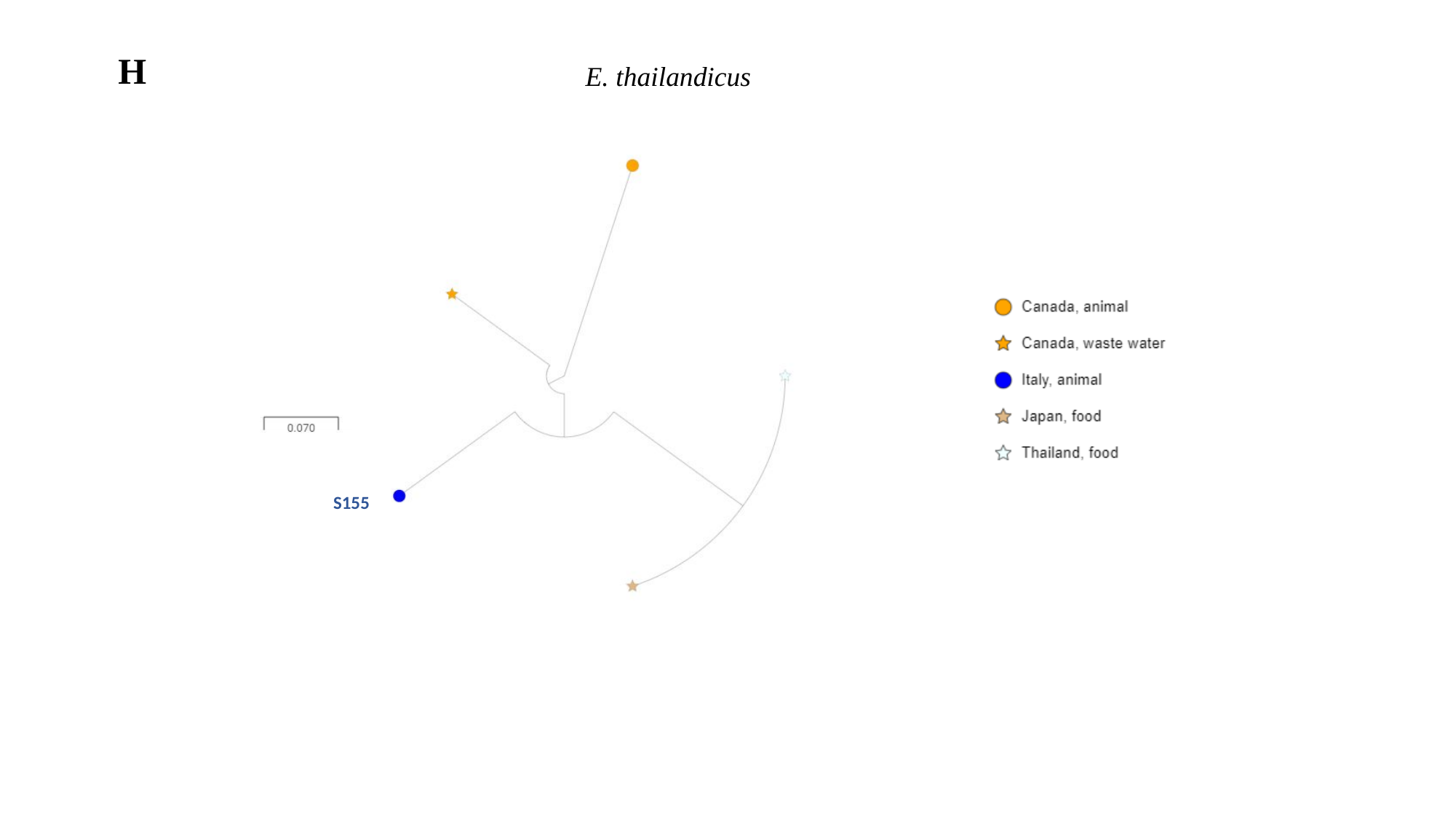

H
E. thailandicus
S155

## Slide 9
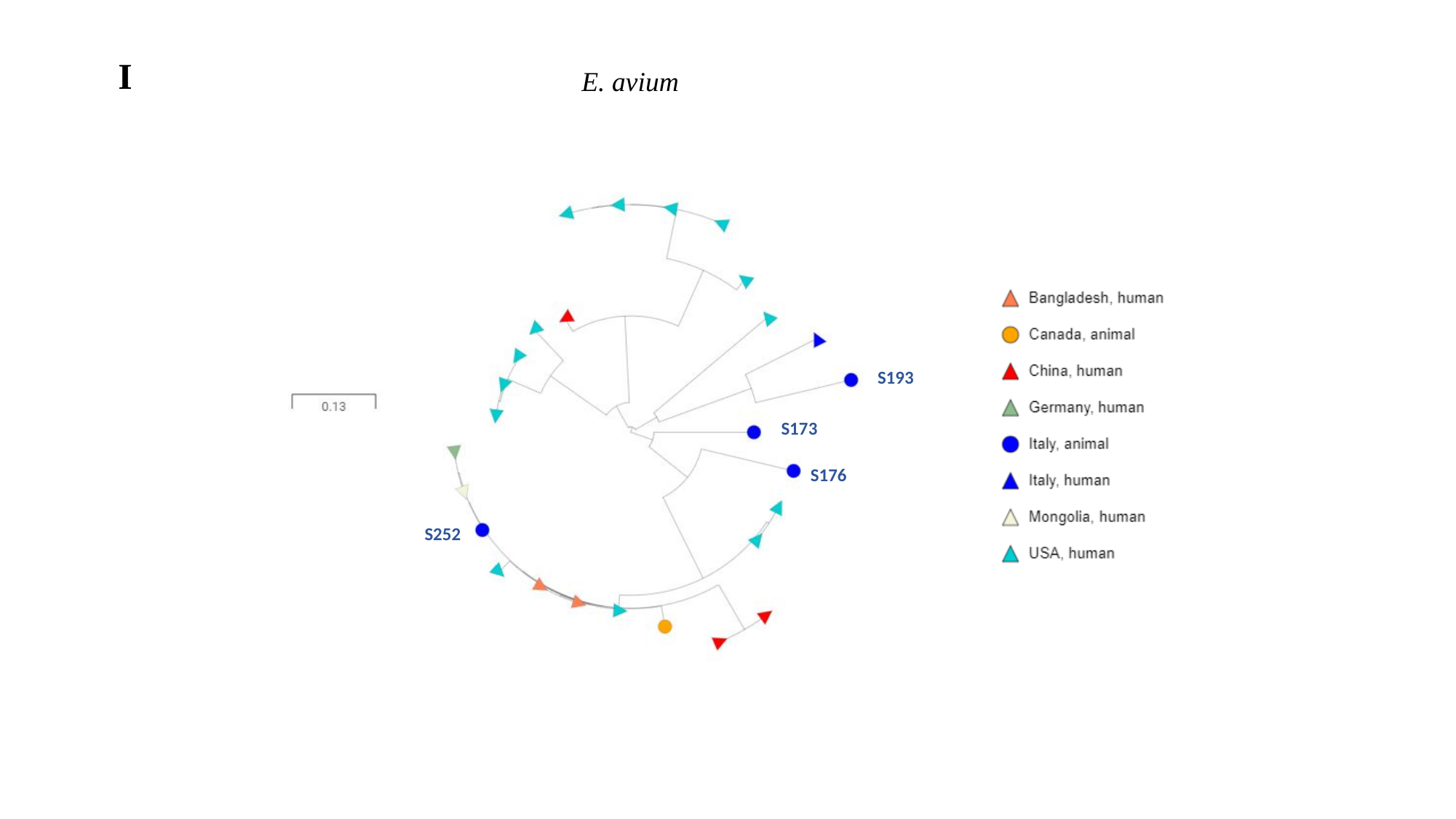

I
E. avium
S193
S173
S176
S252

## Slide 10
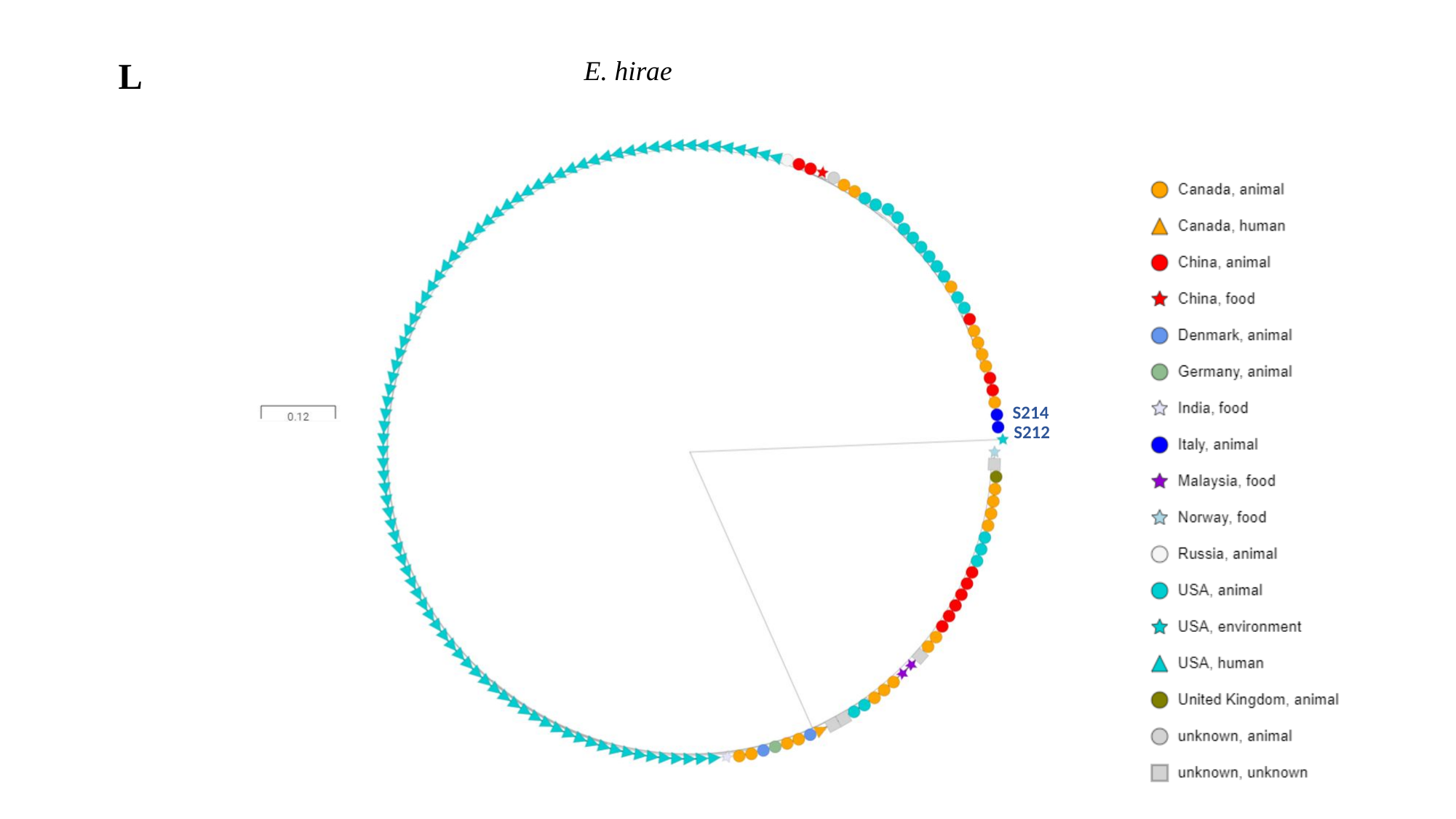

L
E. hirae
S214
S212

## Slide 11
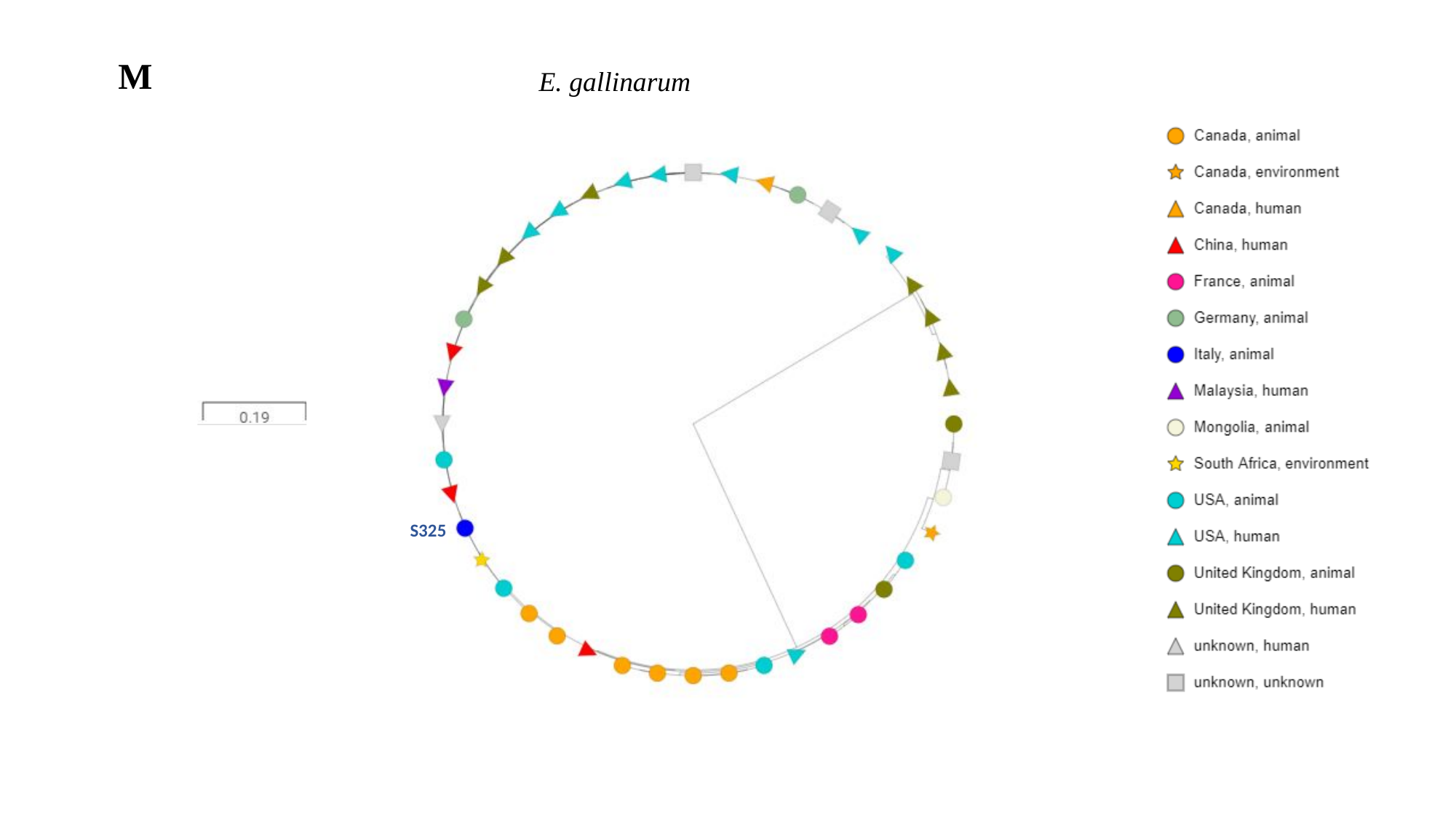

M
E. gallinarum
S325
