# Supplementary figures and images for: Detection of Oxazolidinone Resistance Genes and Characterization of Genetic Environments in Enterococci of Swine Origin, Italy
Source: Microorganisms. 2020 Dec 17;8(12):2021. doi: 10.3390/microorganisms8122021 (PMC7766396; doi:10.3390/microorganisms8122021)

## Slide 1
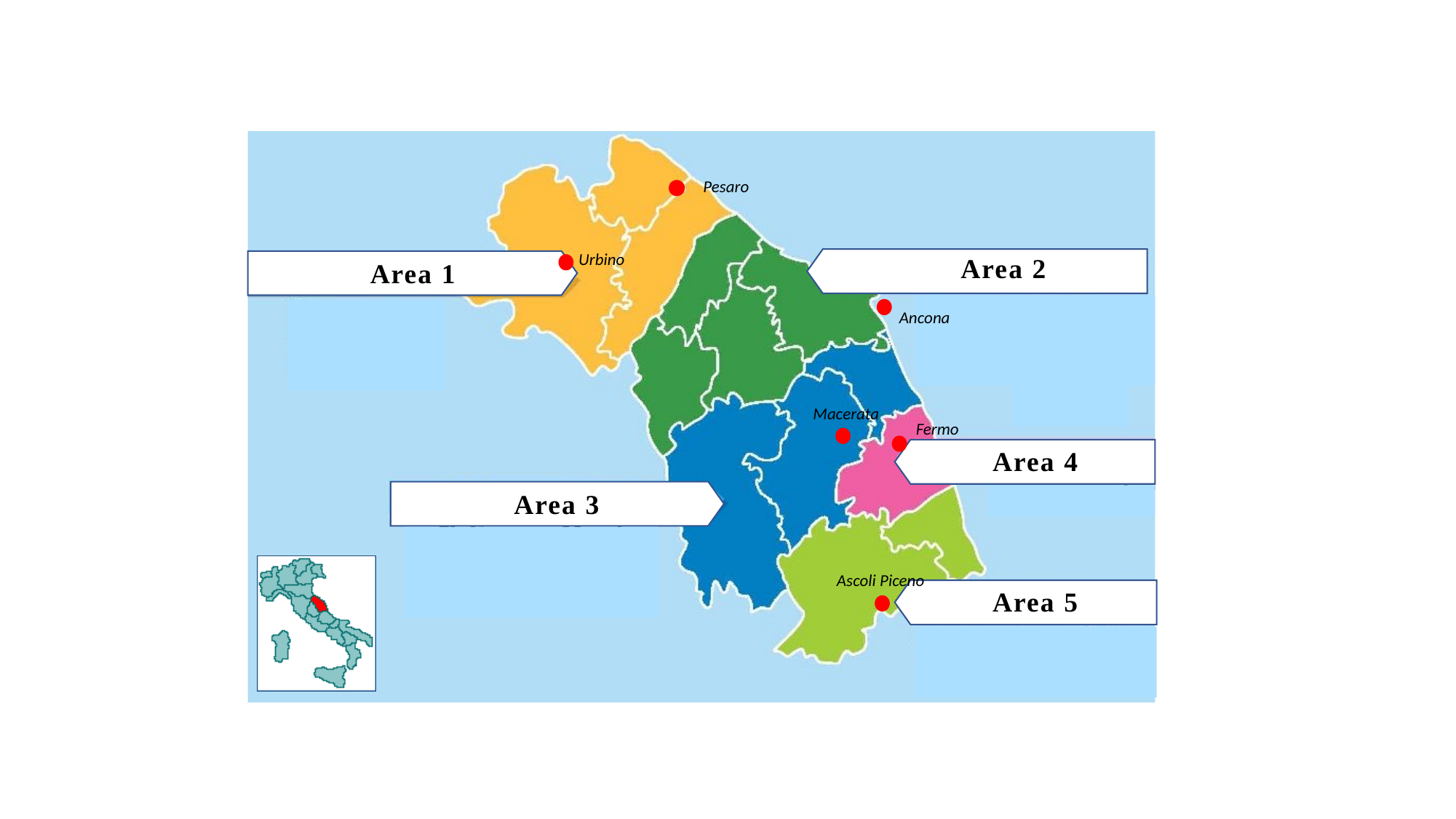

Area 2
N
N
Area 1
Area 4
N
Area 3
N
Area 5
N
Pesaro
Urbino
Ancona
Macerata
Fermo
Ascoli Piceno

Supplement: Supplementary file 1 [file microorganisms-08-02021-s001.zip › Figure S1.pptx]
